# The effects of disruptions in ribosomal active sites and in intersubunit contacts on ribosomal degradation in *Escherichia coli*

**DOI:** 10.1038/srep07712

**Published:** 2015-01-12

**Authors:** Anton Paier, Margus Leppik, Aksel Soosaar, Tanel Tenson, Ülo Maiväli

**Affiliations:** 1 Institute of Technology, Univeristy of Tartu, Nooruse 1, Tartu, 50411, Estonia; 2 Institute of Molecular and Cellular Biology, University of Tartu, Riia 23, Tartu, 51010, Estonia

## Abstract

Although ribosomes are very stable under most conditions, ribosomal degradation does occur in diverse groups of organisms in response to specific stresses or environmental conditions. While non-functional ribosome decay (NRD) in yeast is well characterized, very little is known of the mechanisms that initiate ribosomal degradation in bacteria. Here we test ribosome degradation in growing *Escherichia coli* expressing mutant ribosomes. We found that mutations in the 16S rRNA decoding centre (G530U and A1492C) and 23S rRNA active site (A2451G) do not lead to ribosomal degradation. In contrast, 23S rRNA mutation U2585A causes degradation of both the large and small ribosomal subunits in *E. coli*. We further tested mutations in 23S rRNA, which disrupt ribosomal intersubunit bridges B2a and B3. Deletion of helix 69 of 23S rRNA and the point mutation A1912G in the same helix did not destabilize ribosomes, while expression of mutations A1919G in H69 and A1960G in H71 led to degradation of both mutant and wild-type ribosomes. Our results suggest an actively induced mechanism requiring *de novo* protein synthesis for ribosomal degradation in *E. coli*, which degrades both structurally inactive and active ribosomes.

When a cell divides, it needs to double the mass of its constituent molecules. The rate-limiting factor for cell growth in unicellular organisms is protein synthesis and, more specifically, the concentration of active ribosomes^1^,^2^. In exponentially growing bacteria up to 98% of RNA consists of rRNA and tRNA, and the number of ribosomes *per* cell increases with increasing culture growth rates from about 20 000 to 70 000^3^.

The *Escherichia coli* ribosome is a very large (2.4 MDa), intricately structured, two-subunit ribonucleoprotein consisting of three rRNAs and about 54 different r-proteins, whose assembly is catalyzed by about 20 extra-ribosomal proteins^4^. It would thus be reasonable to assume that the cost of making such a large molecular structure would justify a long and stable life for the ribosome. Although ribosomal RNA has long been the epitome of stable RNA and is indeed stable during constant growth^5^, there are numerous accounts of significant amounts of ribosomal degradation of both misassembled ribosomal particles and of fully assembled ribosomes in response to various cellular stresses in bacteria (reviewed in Refs. 6,7). Degradation of ribosomes has also been observed during the slowing of culture growth that precedes the onset of the stationary growth phase^5^.

A growing body of evidence suggests that degradation of ribosomal RNA in *E. coli* is a frequent and complex phenomenon, which is likely to be triggered and regulated by dedicated factors. While the endonuclease(s) responsible for initiation of ribosomal degradation remain unidentified, ribosomal subunit dissociation and the ensuing increased availability of the RNA-rich intersubunit surfaces was, based on subjecting dissociated ribosomal subunits to cellular lysates, proposed to act as a trigger for ribosomal degradation^8^. In contrast, a recent paper suggests that it is the assembly of structurally defective 30S subunits into 70S ribosomes that enables the YbeY endonuclease to initiate ribosomal degradation^9^. As a consequence, both the defective 30S subunit and the associated 50S subunit are degraded.

In yeast, degradation of specific ribosomal rRNAs can be triggered by the mutational inactivation of the affected RNA^10^. Mutations in the 25S rRNA peptidyl transferase centre, or PTC, (A2451G or U2585A in *E. coli* nomenclature) result in degradation of 26S rRNA, but not 18S rRNA. Conversely, mutations in the 18S rRNA decoding centre, or DCC, (G530U and A1492C) lead to degradation of 18S rRNA while leaving 25S rRNA intact. Because, unlike in yeast, expressing plasmid-borne ribosome inactivating active site mutations in the context of chromosomally encoded wild-type ribosomes do not generally lead to a reduced fraction of mutant over chromosomal rRNA in *E. coli*, such mutants have been considered stable in *E. coli*^10^.

In the present work we ask whether disrupting ribosomal intersubunit contacts or ribosomal active sites by mutations can induce ribosomal degradation. We study how ribosome-inactivating mutations (A2451G and U2585A) in the 23S rRNA PTC and in the 16S rRNA DCC (G530U and A1492C) affect degradation of mutant and wild-type rRNA. In addition, we study the effects of mutations in the intersubunit bridges B2a (A1912G, A1919G and ΔH69) and B3 (A1960G) of the domain IV of 23S rRNA, which have been shown to structurally destabilize 70S ribosomes *in vivo* and *in vitro*^11^,^12^,^13^.

## Results

### Experimental setup

**Figure 1:**
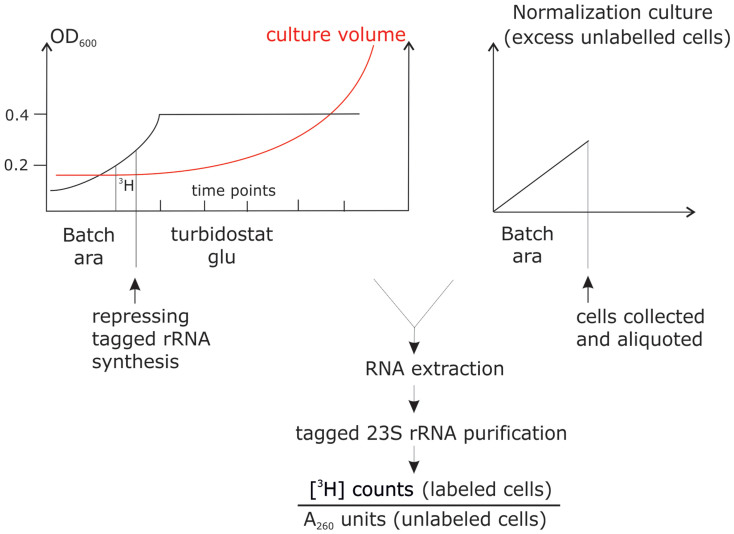
A scheme of the experimental setup. The study culture expressing tagged ribosomes from an arabinose-inducible promoter is pulse-labeled with [^3^H]-uridine in batch, tagged ribosome expression is subsequently repressed by medium switch to glucose-containing LB, and the culture is transferred to a turbidostat for constant-rate growth. Subsequently samples are collected from the study culture at designated time points and each sample is mixed with 20 ml of unlabeled normalization-culture cells expressing tagged ribosomes. Tagged rRNAs are purified and data expressed as normalized specific activities. A_260_, absorbance at 260 nm; ara, arabinose; gluc, glucose.

**Figure 2:**
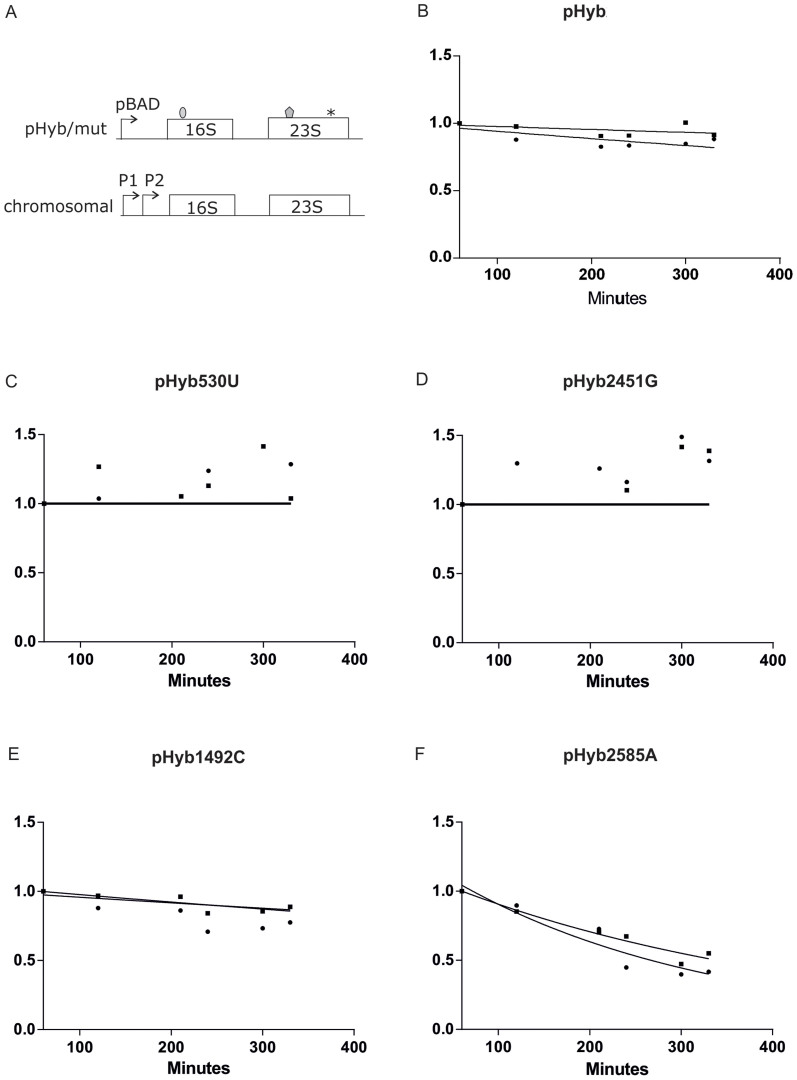
Degradation of tagged 23S rRNAs and 16S rRNAs in the presence of ribosomal active site mutants. Cultures expressing pHyb (panel B), pHyb530U (panel C), pHyb2451G (panel D), pHyb1492C (panel E), and pHyb2585A (panel F) were grown in in batch culture under the presence of inducer until OD_600_ = 0.2, pulse-labeled with [H^3^] uridine, transferred to repressor-containing fresh LB medium and grown in turbidostat. (A). A scheme of rRNAs used in respective experiments. RNA tags in 16S rRNA and 23S rRNA are shaded and the point mutation is depicted as an asterix. (B). pHyb “wild-type” control (n = 3), (C). G530U mutation in pHyb 16S rRNA gene (n = 2), (D). A2451G mutation in pHyb 23S rRNA gene (n = 2), (E). A1492C mutation in pHyb 16S rRNA gene (n = 3), (F). U2585A mutation was cloned into the pHyb 23S rRNA gene (n = 3). Sample size (n) refers to the number of independent biological replicates. Circles and squares denote mean values of 23S rRNA and 16S rRNA data, respectively. Specific activities obtained in each individual time course were normalized and ensuing data fitted to a one phase exponential degradation curve as described in Materials and Methods. The x-axis shows time in minutes and Y-axis shows normalized specific activity ([H^3^] counts/OD_260_).

Ribosomal stability measurements were conducted under constant growth in turbidostat as described in^5^ (Figure 1). To express the mutationally inactivated rRNAs in the background of chromosomally encoded wild-type ribosomes, we utilized a plasmid-borne expression system containing the rrnB rRNA operon under the arabinose-inducible pBAD promoter (Figure 2A). The expression from this promoter can be repressed by replacing arabinose with glucose in the growth medium. The plasmid-encoded 23S rRNA gene contains a streptavidin-binding RNA aptamer and the 16S rRNA gene contains a MS2 binding aptamer^5^.

We transiently expressed aptamer-containing ribosomal RNAs in arabinose containing growth media (LB) and further pulse-labeled the ribosomes with [^3^H]-uridine. At the point of repression the tagged ribosomes constituted about 15% of total cellular ribosomes (Supplemental Figure S1). After repressing tagged ribosome synthesis we can follow the degradation of a defined population of ribosomes, which are synthesized in a short (45 minute) time window of our choosing, corresponding to the [^3^H]-uridine pulse (Figure 1). The continuous cellular production of non-radioactive chromosomally encoded ribosomes allows us to study the degradation of aptamer-tagged and radioactively labeled ribosomes in normally growing cells. After repression of aptamer-containing rRNA synthesis by switching to non-radioactive glucose containing growth medium, cultures were transferred to turbidostat for continuous growth, samples were collected at designated time points, and the affinity tagged rRNAs were purified (see Methods for detail). Affinity-purification removes the non-tagged chromosomally encoded ribosomes from the analysis and allows us to study ribosomal degradation by ignoring the dilution of tagged ribosomes by newly made chromosomally encoded ribosomes. To account for inter-sample variation in cell lysis and purification efficiencies, all radioactively labeled samples were mixed prior to lysis with an equal amount of non-radioactive normalization culture expressing tagged ribosomes. This normalization culture is in large excess and thus allows us to use the A_260_ units to normalize [^3^H] counts of purified labeled rRNAs. Results were expressed as normalized specific activities (radioactive counts, which derive from the study culture, divided by tagged RNA concentrations, which mostly represent the excess normalization culture). Because rRNA is the major component of ribosomes and serves as the scaffold for r-proteins, its degradation effectively measures the degradation of ribosomes.

Although both the 23S rRNA and 16S rRNA purification tags are spatially well separated from the active site mutations, we cannot fully exclude synergistic effects between them, which could affect ribosomal degradation patterns. However, cloning of the ribosomal active site point mutations and the RNA affinity purification tags into separate plasmids allowed us by co-expressing both plasmids to study the trans-effects of ribosome-inactivating mutations on the stability of translationally fully active tag-containing ribosomes. We found that separating the purification tags and point mutations into different rRNA molecules does not affect degradation of cellular ribosomes, speaking against synergistic effects between point mutations and purification tags in our experimental system (see section “Mutant ribosomes induce degradation of wild-type ribosomes” below).

### Degradation of ribosomes containing mutations in their active sites

To test whether *E. coli* has nonfunctional rRNA decay (NRD) similar to yeast^10^, we introduced the same ribosome-inactivating mutations that were used to demonstrate NRD in yeast into our arabinose controlled pHyb expression system and measured ribosomal degradation during constant growth of *E. coli* in turbidostat. Mutations G530U and A1492C in 16S rRNA have been shown to disrupt activity in the decoding center within the small subunit and mutations in 23S rRNA positions A2451G and U2585A in the peptidyl transferase reaction center within the large subunit of *E. coli* also disrupt activity^14^,^15^,^16^. Expression of all four mutations lead to degradation of the affected ribosomal subunit in yeast^10^.

As was previously seen^5^, the basic tagged pHyb construct gave in our turbidostat experiments rise to stable ribosomes (Figure 2B and Supplemental Figure S2A).Introduction of either the inactivating G530U mutation into the 16S rRNA or A2451G into 23S rRNA of the pHyb construct did not significantly destabilize either 16S or 23S rRNAs (Figure 2C–D, Supplemental Figure S2B–C). In addition, an A1492C mutation in the decoding center of 16S rRNA may have a small destabilizing effect on the ribosomes (Figure 2E, Supplemental Figure 2D), although the temporal duration of the experiments precludes predicting the half-lives of mutant rRNAs with reasonable accuracy. In contrast, a 23S rRNA mutation U2585A in the peptidyl transferase center leads to a marked degradation of both 23S and 16S rRNAs (Figure 2F and Supplemental Figure S2E). The half-life of ribosomes in a mutant 2585A rRNA context was estimated to be between 200–300 minutes (Supplemental Table S1). Because all mutant cultures had similar growth rates in turbidostat (Supplemental Figure S2F), it is unlikely that the increased ribosomal degradation seen in U2585A mutant 23S rRNA expressing cells is caused by a non-specific growth effect.

### Degradation of ribosomes containing mutations in intersubunit contacts

**Figure 3:**
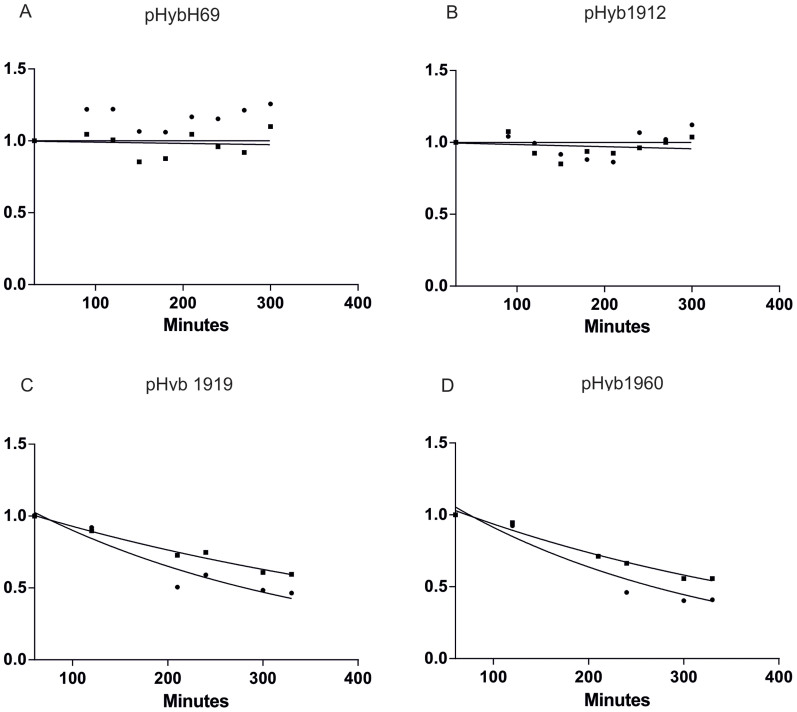
Degradation of tagged 23S rRNAs and 16S rRNAs in the presence of ribosomal intersubunit bridge mutants. (A). deletion of 23S rRNA helix 69 (n = 3), (B). A1912G (n = 2), (C). A1919G (n = 4), (D). A1960G (n = 3) mutations in pHyb 23S rRNA gene. Further details are provided in the legend for Figure 2.

We next introduced mutations into two central intersubunit bridges (B2a and B3), which have been previously shown to confer strong phenotypes, including reducing the fractions of mutant 70S ribosomes and increasing the fractions of mutant 50S subunits in polysomes: A1912G, A1919G, and A1960G^12^,^13^. In addition, we introduced the deletion of helix 69 of the 23S rRNA (ΔH69) which completely abolishes the bridge B2a^11^,^17^. The growth rates of all mutants were similar (Supplemental Figure S3E). Expression of ΔH69 or A1912G mutant ribosomes did not destabilize ribosomes (Figure 3A and B and Supplemental Figure S3A and B). In contrast, when cells expressing A1919G or the A1960G mutations were grown in turbidostat, we found that both 23S and 16S rRNAs were degraded (Figure 3C and D and Supplemental Figure S3C and D). The relative amount of both mutant 23S rRNA and “wild-type” (tag-containing) 16S rRNA were reduced by approximately half over five hours.

Because the MG1655 strain does not produce the 3′ to 5′ exonuclease RNase PH, we retested the effects of mutations A1912G, A1919G, and A1960G in the isogenic MG1655* strain, which contains the gene for RNase PH (a gift from Murray Deutscher). We found that introduction of RNase PH to our system did not change the ribosome degradation patterns observed in our original MG1655 strain (Supplemental Figure S4). This result is consistent with the finding of the Deutscher lab that RNase PH does not participate in rRNA degradation in growing cells^18^. We conclude that disruption of intersubunit contacts can lead to ribosomal degradation by an RNase PH-independent mechanism.

### Mutant ribosomes induce degradation of wild-type ribosomes

All rRNA mutations that cause ribosomal degradation lead to the degradation of both 16S and 23S rRNAs to a similar extent. We next asked whether a prerequisite for degradation of wild-type subunits is their previous association with mutant subunits, or are chromosomally encoded ribosomes that contain no mutant subunits degraded as well?

**Figure 4:**
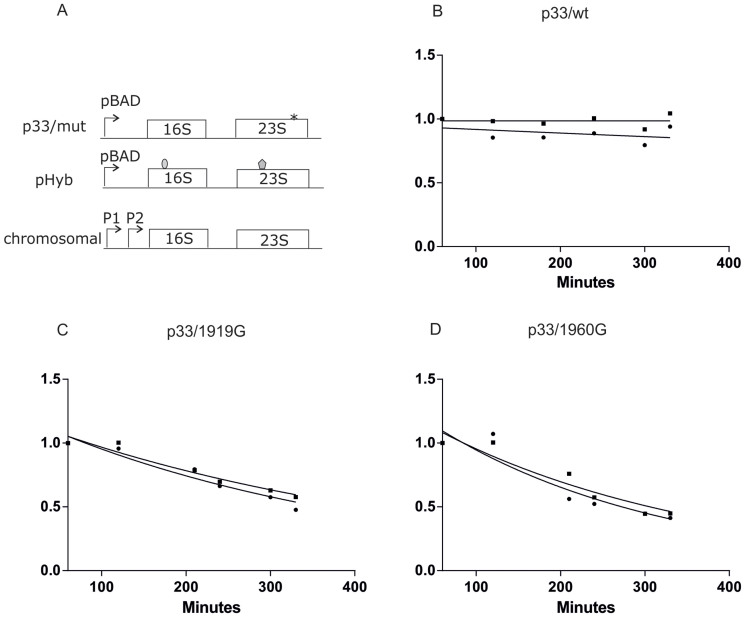
Expression of ribosome-destabilizing mutations in intersubunit bridges leads to degradation of “wild-type” ribosomes. (A). Scheme of rRNAs used in experiments. 16S rRNA and 23S rRNA tags are shaded and the point mutation is depicted as an asterix. (B). p33/wild-type is co-expressed with pHyb (n = 2).(C). p33/1919G is co-expressed with pHyb (n = 3). (D). p33/1960G is co-expressed with pHyb (n = 3). Further details provided in the legend for Figure 2.

In order to see if the presence of the mutant affects the stability of wild-type rRNA, we cloned an untagged rRNA operon containing the intersubunit bridge mutations A1919G or A1960G under the arabinose-regulated pBAD promoter and co-transformed the construct with pHyb. This approach allowed us to isolate and study “wild-type” aptamer-containing rRNAs in the presence of separately expressed ribosomal mutations (Figure 4A).

We found similar degradation to the previous experiments where inactivating point mutations were embedded in tagged rRNAs. A control where the wild-type rnnB rRNA operon was cloned after the pBADara promoter did not exhibit degradation (Figure 4B and Supplemental Figure S5A). In contrast, expression of both the A1919G and 1960G mutant caused degradation of wild-type ribosomes present in the same cells (Figure 4C and D, Supplemental Figure S5B and C). As in previous experiments, there was only a slight reduction in the growth rates of mutant constructs compared to “wild-type” (Supplemental Figure S5D). The extent of degradation of the “wild-type” tagged ribosomes was in both cases similar to degradation observed in the mutant rRNA itself (Figures 3 and 4, supplemental Table 1), thus indicating that while ribosomal degradation was initiated by the expression of the mutant ribosomes, it involves activation of a pathway that is equally adept at degrading mutant and wild-type ribosomes.

### Mutant ribosomes are stable in the stationary growth phase

**Figure 5:**
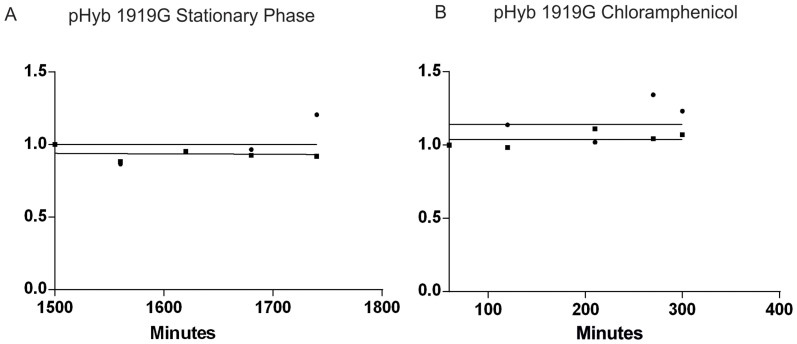
Ribosomes are stabilized in non-growing non-protein synthesizing cells. (A). Ribosomes containing the destabilizing 23S rRNA A1919G mutation are stable in the stationary growth phase. A1919G mutants were grown in inducing conditions (arabinose) to early stationary phase, labeled with [H^3^] uridine for 45 minutes, and switched to exhausted medium with repressor (glucose). Exhausted medium was collected from a culture grown for 24 h before removing the cells by centrifugation. First time point was collected 1 hour after the medium switch. This correspond to 25 hours, or 1500 min as shown on the figure (n = 2). (B). Inhibition of protein synthesis stabilizes the ribosomes. Cell culture expressing the pHyb1919G mutant was grown under inducing conditions until OD_600_ = 0.2, labeled for 30 minutes with [H^3^]-uridine, after which the growth medium was exchanged for glucose-containing LB (repressing medium). 30 minutes after medium switch chloramphenicol (100 µg/ml) was added (n = 4). The first time point was taken 30 minutes after chloramphenicol addition (labeled in the figure as 60 min from medium switch).

The experiments described above were conducted in turbidostat to ensure a constant rate of culture growth. Our previous work showed that wild-type ribosomes, despite being degraded when growth begins to slow down in batch cultures, are stable during both constant growth in turbidostat and no growth in the stationary phase^5^. Therefore, we decided to measure the stability of 23S rRNA mutant A1919G ribosomes that were previously shown to induce the degradation of both the mutant and “wild-type” ribosomes (Figures 3 and 4), during the stationary growth phase of a batch culture. Cells expressing pHyb1919G were labeled with [^3^H]-uridine in the late exponential growth phase, grown into early stationary phase, wherein the inductor was removed by substituting the growth medium for a conditioned medium. The medium switch did not lead to additional growth of the culture (data not shown). The ribosomes were stable during five hours after the medium switch (Figure 5A and Supplemental Figure S6A), which suggests that cell growth is a necessary prerequisite for the degradation of mature ribosomes in *Escherichia coli*.

The stationary growth phase tested in Figure 5A is a product of gradual physiological change and thus an adaptive process. We next asked, whether abrupt inhibition of protein synthesis would be enough to rescue ribosomal degradation. Thus we tested degradation of ribosomes expressing pHyb1919G, which at OD_600_ = 0.2 were switched into fresh medium containing the bacteriostatic protein synthesis inhibitor Chloramphenicol (100 µg/ml). Cloramphenicol inhibits the elongation phase of protein synthesis by binding to the peptidyl transferase active site and thus stalls polysomes on their mRNA templates^19^. As is evident from Figure 5B and Supplemental Figure 6B, inhibition of protein synthesis (and therefore cell growth) by Chloramphenicol led to the stabilization of ribosomes. This result suggests that it is the inhibition of *de novo* protein synthesis, not the physiological state of stationary phase *per se*, which is responsible for stabilizing the ribosomes.

## Discussion

In the current work we have characterized degradation of *E. coli* ribosomes in growing cells expressing specific rRNA mutations in the peptidyl transferase center of the 50S subunit, in the decoding centre of the 30S subunit, and in the central intersubunit bridges B2a and B3. Our results are relevant to the following questions: Does *E. coli* have a similar non-functional ribosome decay program to the yeast NRD^6^,^10^? What triggers mature ribosome degradation in *Escherichia coli*? Is degradation confined to mutationally inactivated ribosomes or are all cellular ribosomes potential substrates for degradation? Does ribosome degradation require specific activation of the degradation machinery?

To compare yeast NRD with ribosomal degradation in *E. coli* we introduced the mutations that led to NRD in yeast in the original work of LaRiviere *et al.*^10^ into *E. coli* rRNA and made use of an experimental system that measures the stability of defined subpopulations of mature cellular ribosomes^5^. To avoid the previously described ribosome degradation that takes place upon slowing of growth in batch cultures, we tested the same inactivating ribosomal mutants in a turbidostat where culture turbidities and thus growth rates are kept constant by continuing monitoring of culture optical density and by automatic addition of fresh medium as needed. While the expression of mutations in both the decoding centre of the 30S subunit (G530U, A1492C) and the 50S subunit (catalytically inactive A2451G) do not cause an NRD-like process in our system, the U2585A mutation in the peptidyl transferase centre did lead to ribosomal degradation. Because the growth rates of different mutants were similar, it is unlikely that the observed ribosomal degradation in the cells expressing U2585A 23S rRNAs is simply due to a general physiological state of the culture. The reason that ribosomal degradation is triggered in the U2585A mutational background while leaving a different PTC mutation intact is, however, a question for future study.

The degradation induced by ribosome inactivating mutations is carried out in *S. cerevisiae* by the NRD process, which only acts on the mutated rRNA. However, in our *E. coli* expression system the U2585A mutation in 23S rRNA led to degradation of both subunits, thus further emphasizing that ribosomal degradation is different in yeast and bacteria.

Zundel *et al.* used *in vitro* evidence to support a theory that ribosomal degradation in *E. coli* is caused by exposure of RNA-rich intersubunit surfaces to cellular RNases^8^. Our *in vivo* data point to a more nuanced picture. While single point mutation in 23S rRNA helices 71 (A1960G) and 69, (A1919G), lead to ribosomal degradation, we were intrigued to find that deletion of the entire helix 69 did not result in ribosomal degradation. The bridge B2a, where H69 is located, plays an important functional role both in subunit association^20^,^21^ and in ribosome recycling. When the subunits separate, the tip of H69 is released from contact with helix 44 of 16S rRNA and becomes fully exposed to the cytoplasm^22^. H69 could thus be an entry point to the 50S subunit for the endonuclease that initiates degradation of the 50S subunit. In this case the deletion of H69 would be expected to block degradation.

To our surprise we discovered that while expression of some of the 23S rRNA mutations that destabilize the central intersubunit bridges B2a and B3, indeed lead to ribosomal degradation, they also lead to the degradation of ribosomes that do not contain any bridge-inactivating mutations (Figure 4). These results are strongly supported by Figures 2 and 3, from which it becomes evident that regardless in which subunit (50S or 30S) the inactivating mutation is located, the other tagged subunit is degraded at a similar rate. As only about 15% of the cellular ribosomes are tagged at the point of repression, it follows that during translation most tagged 50S ribosomes must be associated with non-tagged 30S, and *vice versa*. Thus the presence of mutant ribosomal subunits causes dissociation and degradation of both mutant and wild-type ribosomes.

Meanwhile, co-expressing tagged “wild-type” ribosomes with a degradation-prone mutant G7A form of tRNA^Trp^^23^, or its wild-type variant, does not cause degradation of the ribosomes, thus indicating that ribosomal degradation is not a generic response to overexpression of RNA as such (data not shown).

Our results add to the simple model whereby the trigger for ribosomal degradation is exposure of the RNA-rich intersubunit areas to cytoplasmic RNases. It seems that a specific program, involving *de novo* protein synthesis (see Figure 5B), is activated by the mutant ribosomes and that the ensuing response extends beyond a simple removal of mutationally inactivated subunits. Accordingly, it is noteworthy that the bridge mutations that activated ribosomal degradation in growing cells failed to induce degradation in non- growing cells of the stationary phase (Figure 5A), suggesting that the specific ribosomal degradation mechanism in growing cells is inactive in the stationary phase cultures and cannot be induced by the presence of mutant or otherwise dissociated ribosomes.

A model, whereby degradation of ribosomal subunits is triggered by overexpression of an endonuclease, which then cleaves the RNA-rich intersubunit surfaces normally shielded in the 70S ribosome, would provide an explanation, why mutant and wild-type ribosomes are co-degraded in *E. coli*. Accessible intersubunit surfaces (produced by normal ribosomal recycling or by abnormal structures in mutant ribosomes) in conjunction with increased cellular RNase concentrations would then determine the targets and rates of degradation. This model is consistent with the observed rates of ribosomal degradation, where over 300 minutes and four to five cell divisions are needed to degrade 50% of tagged ribosomes (Figures 2, 3, S2, S3). In contrast, in the much faster and more tightly controlled yeast NRD, ribosome degradation is initiated at the 80S ribosome level and different mutant subunits are degraded by non-overlapping separate mechanisms^6^.

In summary, our study suggests that degradation of mature ribosomes in *Escherichia coli* is functionally different from the NRD program of *Saccharomyces cerevisiae*. Further studies are needed to unambiguously disentangle the molecular mechanisms, including the putative pathways of RNases and specific regulatory mechanisms responsible for ribosome degradation in growing *E. coli*.

## Methods

### Plasmids and strains

#### Ribosome degradation experiment in turbidostat

These experiments were performed essentially as described in^5^. MG1655 *E. coli* cells were transformed with pHyb plasmids and grown in batch at 37°C in LB medium supplemented with 0.3% arabinose and ampicillin for plasmid selection. At OD_600_ = 0.2 thirty μl of 5,6-[^3^H]-uridine (Hartmann, 1 mCi/ml, specific activity 35.6 Ci/mmol) was inoculated into 5 ml culture, which was then grown for a further 45 min at 37°C. Cells were then pelleted by centrifugation at 5 000 rpm for 10 minutes at room temperature and resuspended in 5 ml fresh LB medium supplemented with 0.4% glucose and 1 mM non-radioactive uridine. The 5 ml starting culture was subsequently grown in turbidostat at 37°C where OD_600_ was kept between 0.4 and 0.45 U by inflow of pre-warmed LB, glucose, ampicilin medium. At indicated times, aliquots of volume proportional to the volume of the turbidostat culture, to account for the fresh medium added in order to keep the culture density constant, starting with an initial sample of 200 μl, were collected and stored at −85°C. In control experiments, 500 μl of culture at 37°C was inoculated with 3 μl 5,6-[^3^H]-uridine 30 minutes after shifting the cells to glucose-containing media. Very little ^3^H-uridine was incorporated into the tagged ribosomes when added 30 minutes after repression.

A 200 ml normalization culture with unlabeled LB medium containing 0.2% arabinose was also grown in batch condition until OD_600_ = 0.5. The cells were pelleted and stored at −85°C. Each sample from the study culture was mixed with identical volumes of unlabeled cells from the normalization culture. This was done to minimize inter-sample variability during cell lysis and affinity-purification of tagged rRNAs. After purification of tagged ribosomes we normalized the radioactivity counts from the purified rRNA against absorbance at 260 nm, which came mostly from the excess unlabeled rRNA of the normalization culture.

Before lysis, the pellet from the unlabeled culture was resuspended as equal batches of 450 μl TEN buffer containing 1% Brij and 1% SDS. This suspension was used to resuspend the [H^3^]-labeled cell pellets. RNA was extracted by a 10 minute incubation in equal volume of phenol pH 5.5 at 65°C, followed by extractions with 1:1 phenol:chloroform and chloroform on ice. After ethanol precipitation the RNAs were dissolved in 1 ml LLP buffer (10 mM Tris pH = 7.5, 60 mM NH_4_Cl_2_, 60 mM KCl,10 mM MgCl_2_, 6mM 2-mercaptoethanol) and each sample was divided into two equal parts for 23S rRNA and 16S rRNA purification. 23S rRNAs tagged with streptavidin binding aptamer were incubated overnight with 20 μl Sepharose High Performance Streptavidin sepharose (GE Healthcare), washed 4 times with 800 μl LLP, and incubated overnight with 20 μl (5 mM) biotin (Sigma-Aldrich). After centrifugation, the supernatant containing the streptavidin-tagged 23S rRNAs was extracted. 16S rRNAs carrying the M2S tag were purified by incubation with both 6 µl MS2 protein (titrated for activity) and 150 µl amylose resin (BioLabs), washed with binding buffer (20 mM Tris pH = 7.5, 0.1 M NH_4_Cl, 10 mM MgCl_2_), and placed in a 1.5 ml amylose resin column, washed 5 times with binding buffer and eluted with 300 µl elution buffer (20 mM Tris pH = 7.4, 0.2 M NaCl, 10 mM Maltose). Concentrations of 23S rRNA and 16S rRNA were measured by absorbance at 260 nm. The amount of incorporated [H^3^]-uridine was measured by scintillation counting and normalized for the A_260_ absorbance value of the sample. After RNA extraction and prior to purification, the total radioactivity was measured. The specific activity of each sample was further normalized for the total radioactivity after RNA extraction to account for differences in extraction efficiency. To enhance the quantitative comparability between independent replications of experiments the specific activities of each individual time course were further normalized to the mean specific activity of that time course. In the interest of easy comparability at the inter-experiment level the resulting time-curves were normalized to start at one. Single exponential fitting of the degradation curves (one-phase decay model, plateau value set at zero) was done in GraphPad Prism *vers*. 6.

#### Experiment with co-transformed mutant and “wild-type” plasmids

pBAD33 plasmid^24^ was double-restricted with KpnI and HindIII and untagged rrnB operon from a pBAD plasmid (BamHI/XhoI fragment) was cloned into it. 1919G and 1960G mutations were introduced by cloning from the mutant plasmids^11^. Each construct was co-transformed with the pHyb plasmid using chloramphenicol and ampicillin selection. As a control, a construct in which an untagged wild-type rrnB operon was cloned in the pBAD33 plasmid was co-transformed with the pHyb plasmid.

## Author Contributions

A.P. contributed to designing the study, performed the experiments, analyzed the results, and participated in the writing of the paper. M.L. participated in cloning experiments and measured tagged RNA expression levels, A.S. participated in mutagenesis experiments, T.T. participated in designing of the study and in writing of the paper, Ü.M. participated in designing the study, in data analysis, and in writing of the paper.

## Supplementary Material

Supplementary Information Supplemental information
