# Supplementary material for: The effects of disruptions in ribosomal active sites and in intersubunit contacts on ribosomal degradation in Escherichia coli
Source: Sci Rep. 2015 Jan 12;5:7712. doi: 10.1038/srep07712 (PMC4289901; doi:10.1038/srep07712)
Supplement: Supplementary Information — Supplemental information [file srep07712-s1.pdf]

Supplementary Material

for

The effects of disruptions in ribosomal active sites and in intersubunit contacts on ribosomal degradation in *Escherichia coli*

by

Anton Paier, Margus Leppik, Aksel Soosaar, Tanel Tenson, and Ülo Maiväli.

Table S1. Summary of the regression analysis statistics for exponential fitting.

Mutant	Figure	R^2	half-life (minutes)	95% CI*
wt 23S	1A	0.04	ND	ND
wt 16S	1A	0.00	ND	ND
530U 23S	1B	0.00	ND	ND
530U 16S	1B	0.00	ND	ND
1492C 23S	1C	0.45	622	391-1521
1492C 16S	1C	0.35	1221	720-4004
2451G 23S	1D	0.00	ND	ND
2451G 16S	1D	0.00	ND	ND
2585A 23S	1E	0.83	194	155-258
2585A 16S	1E	0.90	280	237-342
1912 23S	2A	0.00	ND	ND
1912 16S	2A	0.00	ND	ND
delta H69 23S	2B	0.00	ND	ND
delta H69 16S	2B	0.00	ND	ND
1919 23S	2C	0.65	213	161-317
1919 16S	2C	0.83	335	296-445
1960 23S	2D	0.78	193	157-249
1960 16S	2D	0.92	291	251-345
p33/1919 23S	3A	0.92	278	241-330
p33/1919 16S	3A	0.90	321	278-398
p33/1960 23S	3B	0.75	188	142-278
p33/1960 16S	3B	0.79	221	172-310
p33/wt 23S	3C	0.11	ND	ND
p33/wt 16S	3C	0.00	ND	ND
IF3 23S	4	0.85	880	680-1246
IF3 16S	4	0.28	ND	ND
1919 SPh23S	5	0.00	ND	ND
1919 SPh 16S	5	0.07	ND	ND

95% CI – 95% confidence intervals for the half-lifes calculated from the exponential fit.
ND – not determinable due to insufficient temporal duration of experiments

Figure S1

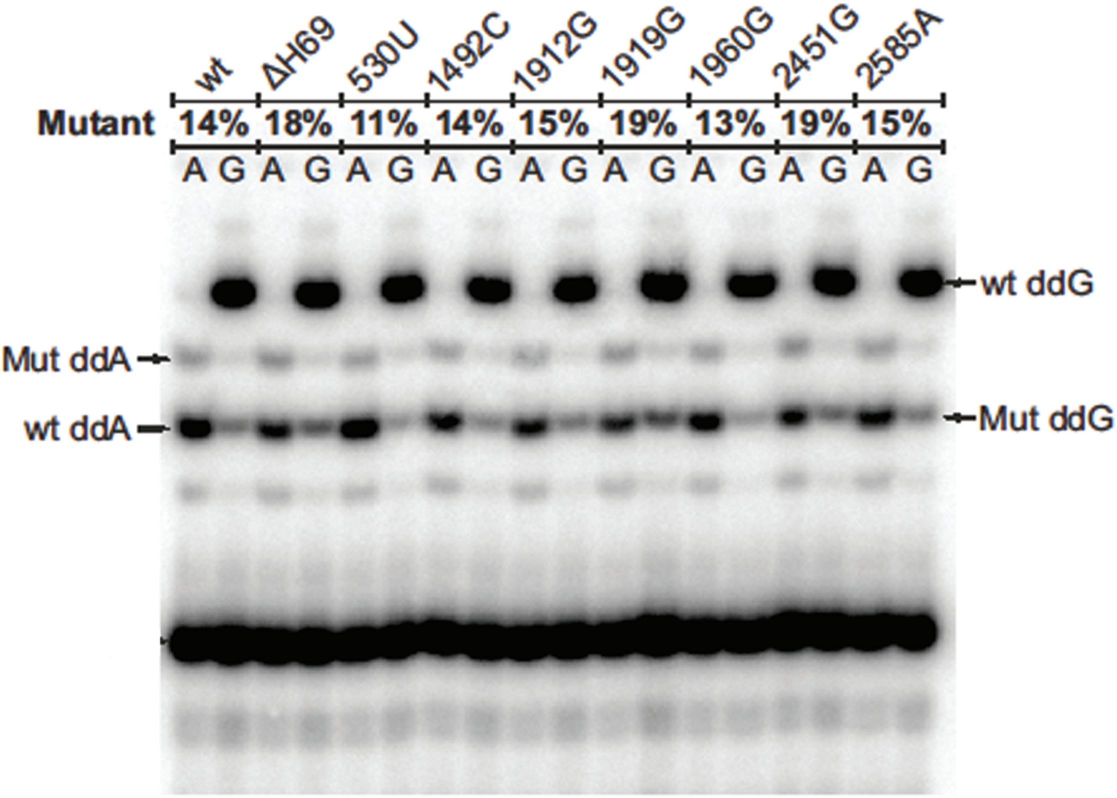

Figure S1. Alalysis of 23S tag in WT and mutant rRNA by reverse transcriptase-directed primer extension was done as described in Liiv, A. and Remme, J, J. Mol. Biol., 1998, vol. 276, pp.537-545. rRNA was extracted with hot phenol, chlorophorm/phenol and chlorophorm. Primer sequence was CAA AAG GTA CGC AGT CAC ACG C. Bands corresponding to tagged and untagged 23S rRNAs are labeled by arrows. Relative proportion of tagged RNA to total cellular 23S rRNA (mutant) is shown as percentages.

Figure S2

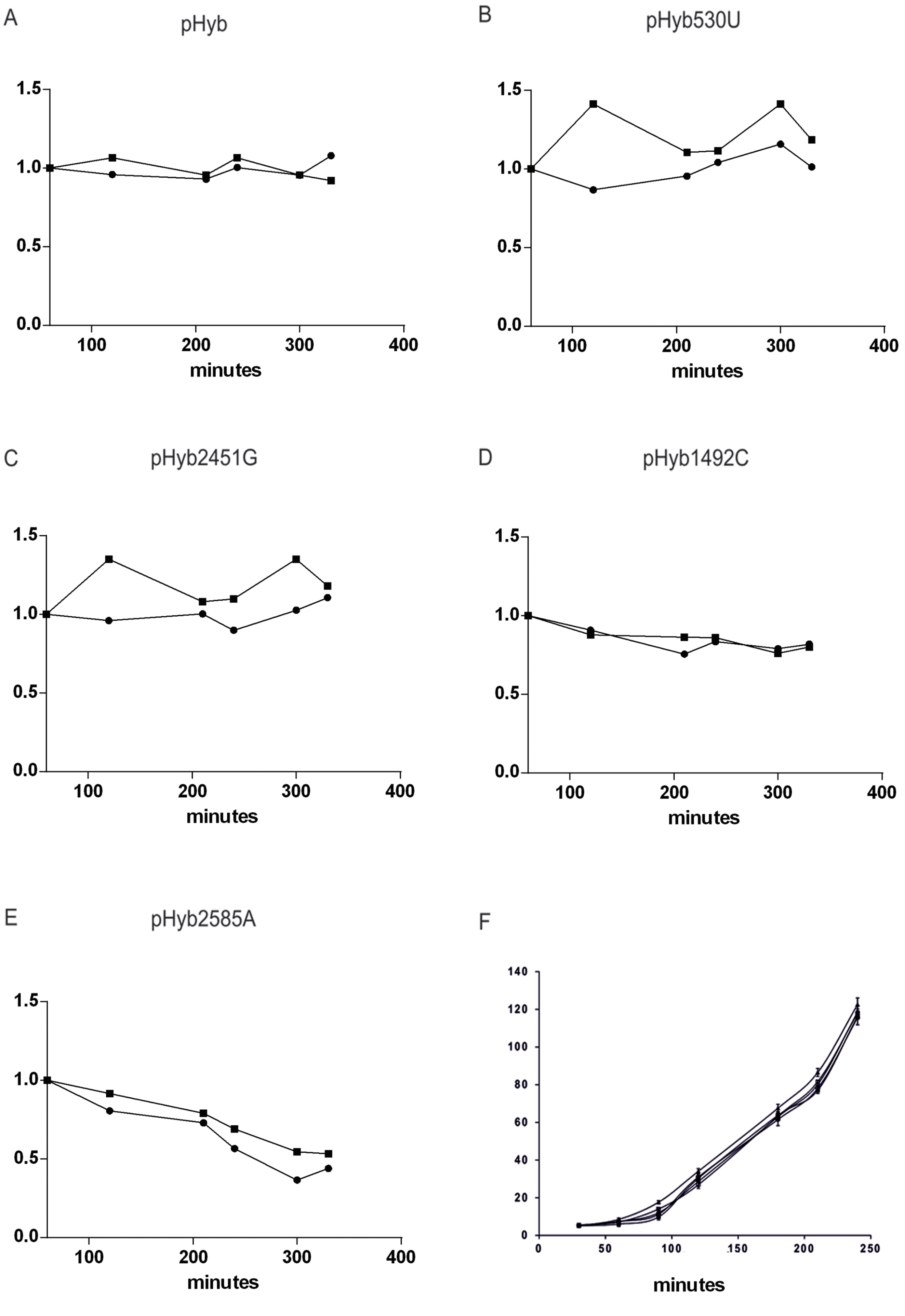
A
pHyb
B
pHyb530U
C
pHyb2451G
D
pHyb1492C
E
pHyb2585A
F
1.5
1.0
0.5
0.0
100
200
300
400
minutes
140
120
100
80
60
40
20
0
50
150
250

Figure S2. Representative experiments of degradation of tagged 23S rRNAs and 16S rRNAs in the presence of ribosomal active site mutants (companion to Figure 2). See Figure 2. legend for details. Circles denote 23S rRNA and squares 16S rRNA. A. pHyb, B. pHyb530U, C. pHyb2451G, D. pHyb1492C, E. pHyb2585A. Panel F shows growth of the cultures in turbidostat where growth rates are controlled by adding fresh LB medium. Y-axis shows total volumes of the cultures in ml. X-axis shows time in minutes. Triangles denote pHyb, diamonds pHyb2585A, open squares pHyb2451G, filled squares pHyb530U and circles pHyb1492C.

Figure S3

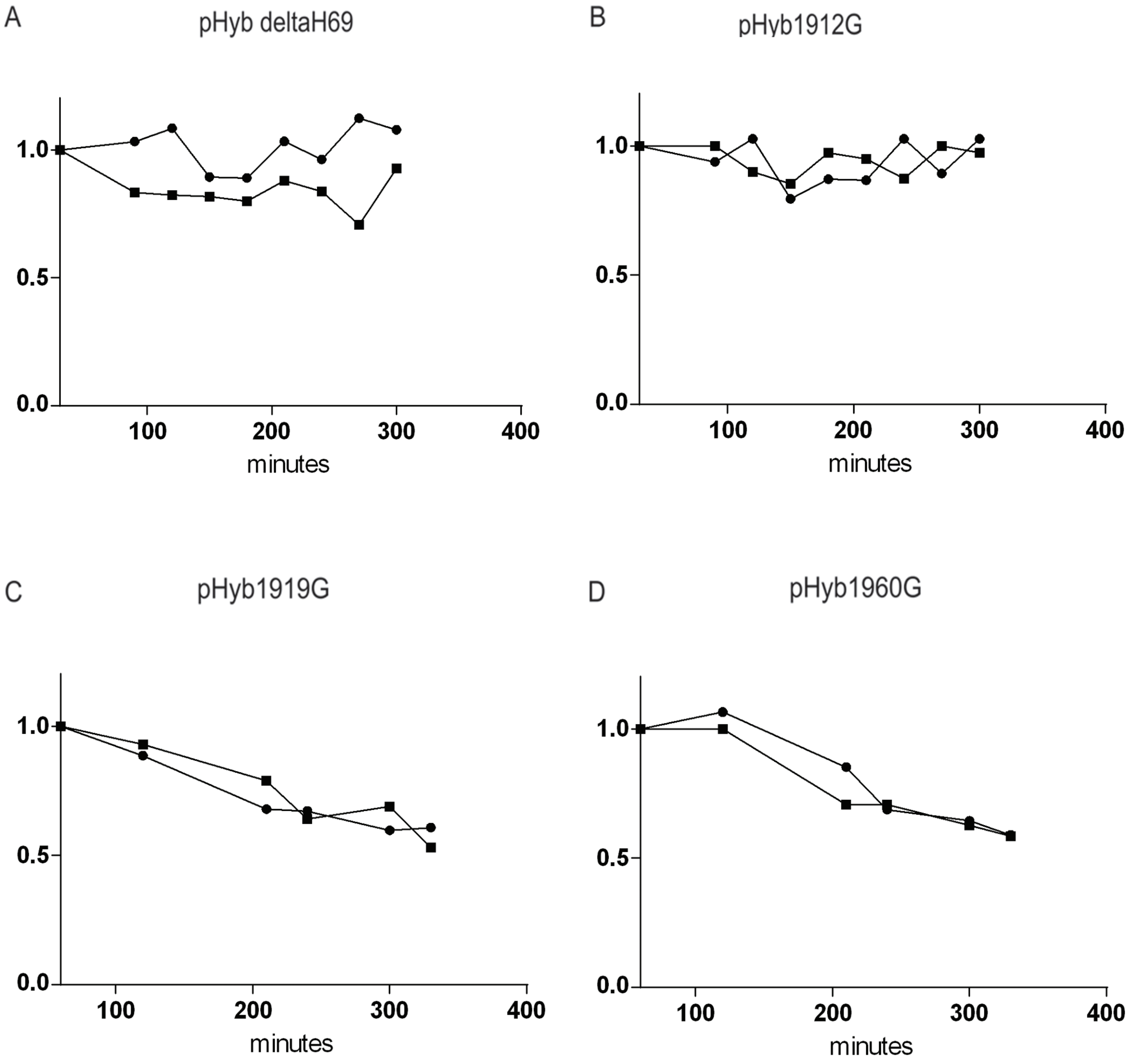

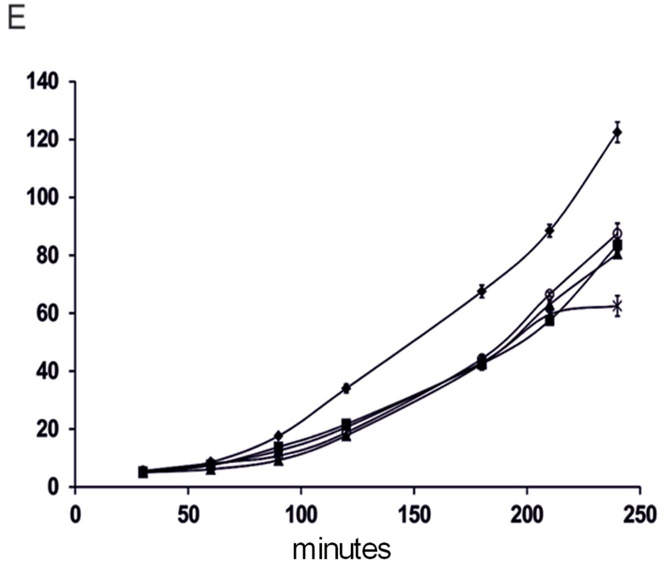

Figure S3. Representative experiments of stabilities of tagged 23S rRNAs and 16S rRNAs in the presence of ribosomal intersubunit bridge mutants (companion to Figure 3). See Figure 2. legend for details. A. pHybΔH69, B. pHyb1912G, C. pHyb1919G, D. pHyb1960G. Triangles denote 23S rRNA and squares 16S rRNA. Panel E shows growth of the cultures in turbidostat where growth rates are controlled by adding fresh LB medium. Y-axis shows total volumes of the cultures in mL. Diamonds denote pHyb, open circles pHyb1912G, squares pHyb1919G, triangles pHyb1960G and crosses pHybΔH69.

Figure S4

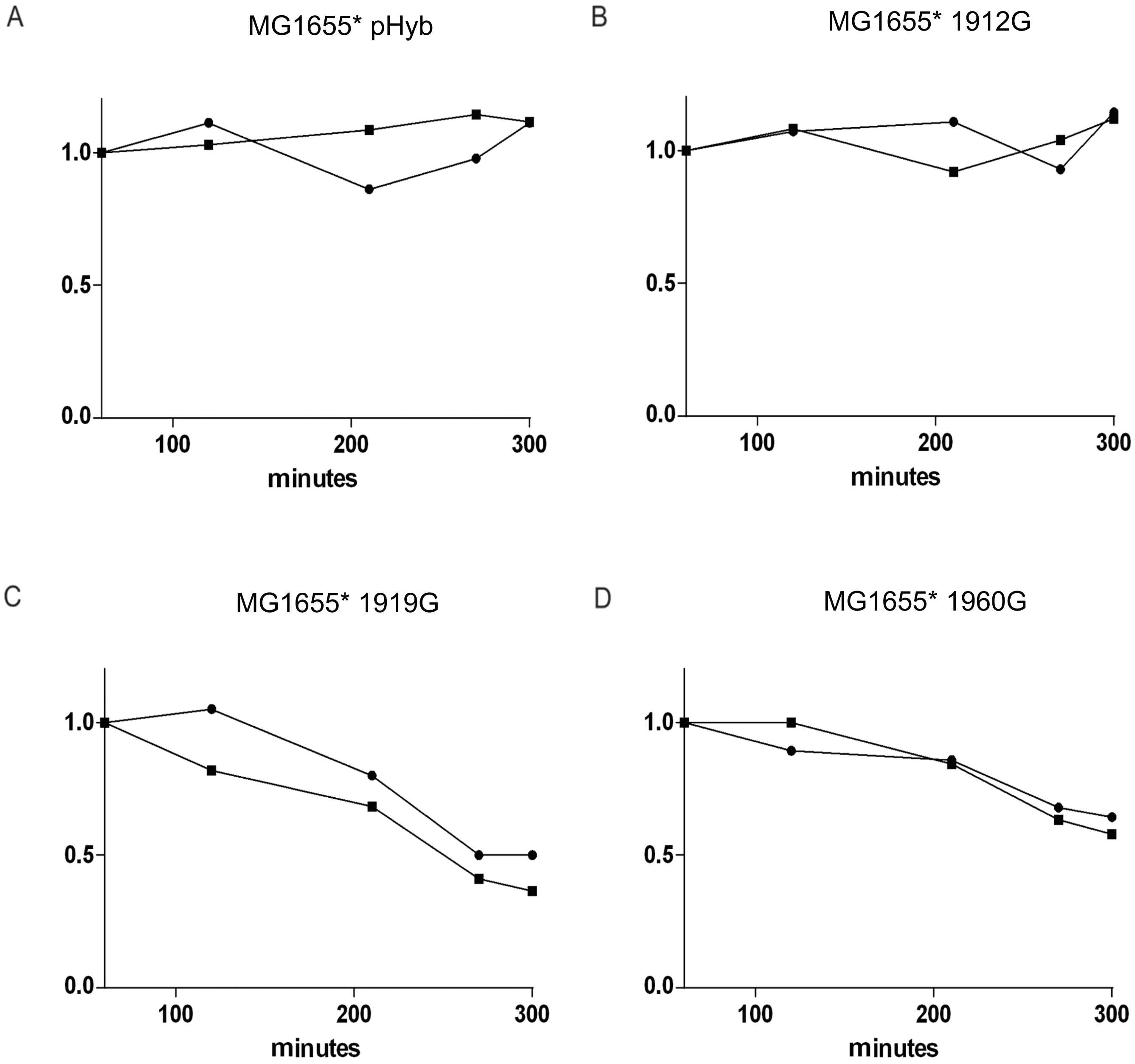

Figure S4. Degradation of tagged 23S rRNAs and 16s rRNAs in the presence of ribosomal active site mutants in MG1655* strain containing the gene for the RNase PH protein. See Figure 2 legend for details. A. pHyb, B. pHyb1912G, C. 1919G, D. 1960G. Circles and squares denore 23S and 16S RNAs respectively.

Figure S5

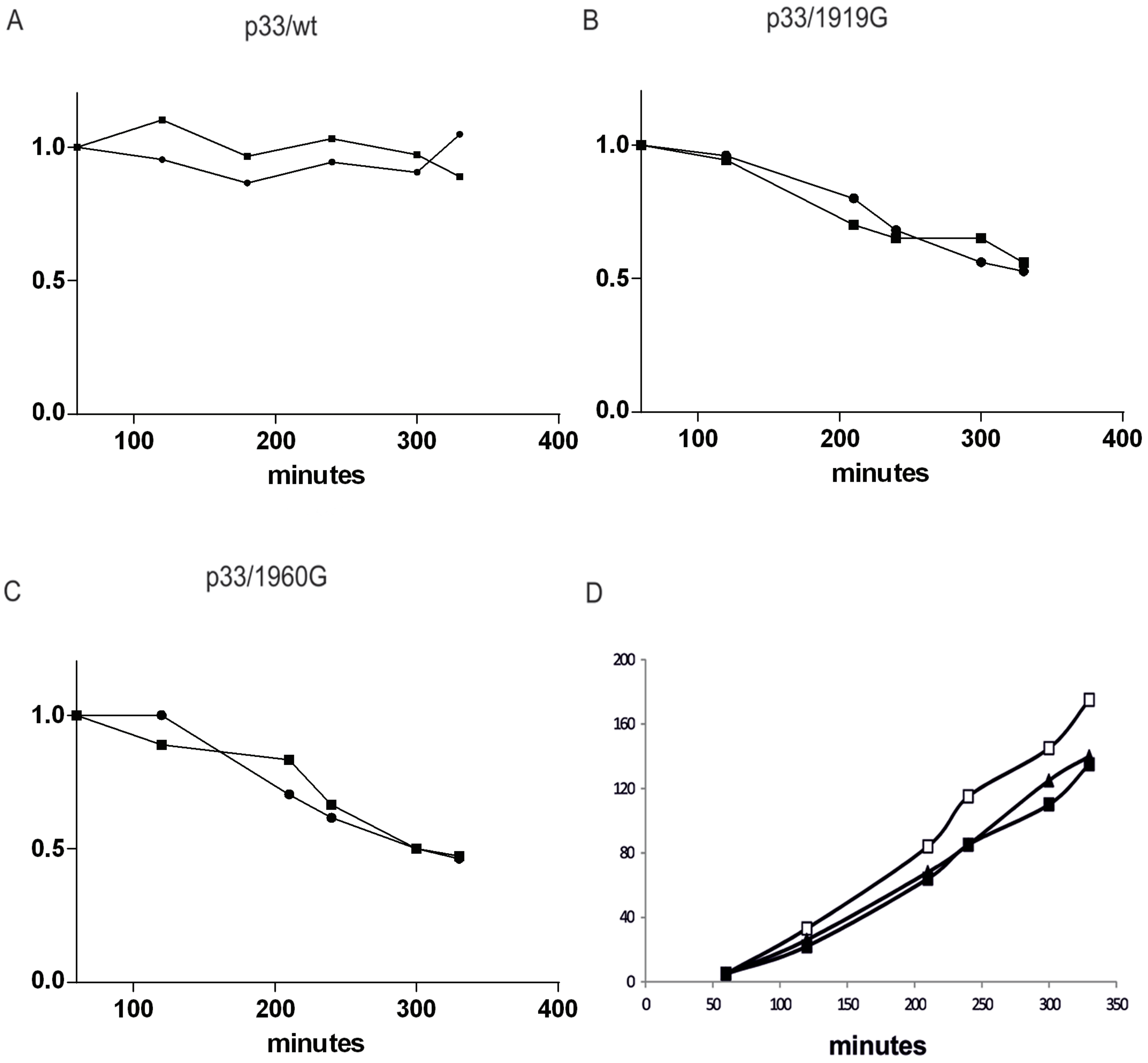

Figure S5. Expression of ribosome-destabilizing mutations in intersubunit bridges leads to degradation of “wild-type” tagged ribosomes. Results from representative experiments are shown (companion to Figure 4). A. p33/1919G is coexpressed with pHyb. B. p33/1960G is coexpressed with pHyb. C. p33/wt is coexpressed with pHyb. D. Growth of the cultures in turbidostat where growth rates are controlled by adding fresh LB medium. Y-axis shows total volumes of the cultures in mL.

Figure S6

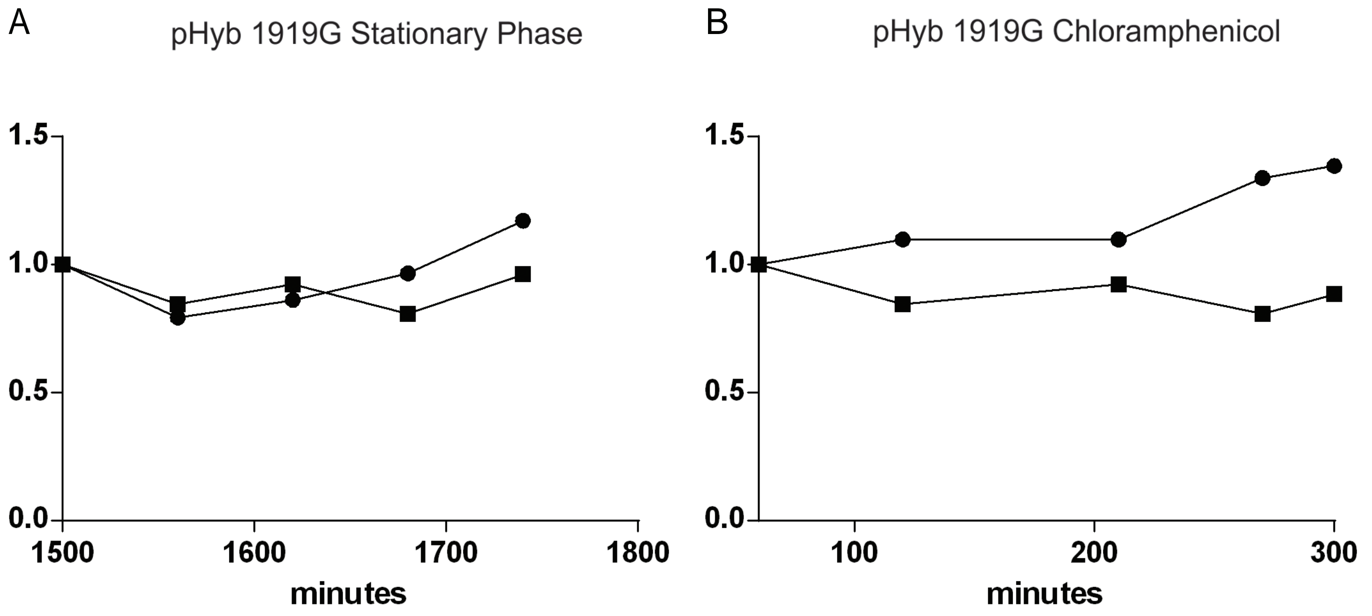

Figure S6. Ribosomes containing the destabilizing 1919G intersubunit bridge mutation are stable in the stationary growth phase. A. A representative experiment is shown. Cell cultures were grown to early stationary phase, followed by 45 minutes pulse labeling with H^3-uridine and medium-switch to depleted LB medium. After that, cultures were shaken at 37 ºC in batch and indicated time points were taken. B. Ribosomes from cell culture grown with chloramphenicol (100 µg/ml) added 30 minutes after medium switch are stable. A representative experiment is shown.
